# Evaluating long-term MRSA colonization and household spread: Insights from a community-based study

**DOI:** 10.1016/j.bjid.2025.104518

**Published:** 2025-02-26

**Authors:** Taniela Marli Bes, Robson Eduardo Soares, Roberta Ruedas Martins, Lauro Perdigao-Neto, Diego Mongelos, Luisa Moreno, Andrea Moreno, Gerson Salvador de Oliveira, Silvia Figueiredo Costa, Anna Sara Levin

**Affiliations:** a Hospital das Clínicas da Universidade de São Paulo, Divisão de Doenças Infecciosas, São Paulo, SP, Brazil; b Instituto de Medicina Tropical da Universidade de São Paulo, São Paulo, SP, Brazil; c Universidade de São Paulo, Faculdade de Medicina, Hospital das Clínicas, Grupo de Controle de Infecções Hospitalares, São Paulo, SP, Brazil; d Universidade de São Paulo, Faculdade de Medicina Veterinária e Zootecnia, São Paulo, SP, Brazil; e Universidade de São Paulo, Faculdade de Medicina, Hospital Universitário, São Paulo, SP, Brazil; f Universidade de São Paulo, Faculdade de Medicina, Hospital das Clínicas, Instituto de Medicina Física e de Reabilitação, São Paulo, SP, Brazil

**Keywords:** MRSA colonization, Household transmission, Community-acquired MRSA (CA-MRSA), Spontaneous clearance, Antibiotic resistance, Infection control, Nasal carriage

## Abstract

Methicillin-Resistant *Staphylococcus Aureus* (MRSA) is commonly transmitted among hospitalized patients through direct contact or contaminated objects. However, the dynamics of household transmission of MRSA remain unclear, posing challenges for effective prevention. This study evaluates the persistence of MRSA colonization in asymptomatic carriers over a period of at least 17-months and examines the potential for intra-household transmission. We conducted home visits to seven families, each with at least one MRSA-colonized member, to collect nasal swabs from all household members. Phenotypic and genotypic profiles of the isolates were determined through culture, antimicrobial susceptibility testing, and PCR. We compared these new samples with previous samples from a recent study involving the same individuals to assess spontaneous clearance of MRSA. A total of 25 samples were collected, with 56 % (14) identified as *S. aureus* and 44 % (11) as non-S. aureus; among the *S. aureus* isolates, four were MRSA. We observed spontaneous clearance of MRSA in six of the original cases. Unexpectedly, there was limited intra-household transmission of MRSA, although all families with MRSA colonization had at least one member with a history of skin disease. In the family where colonization persisted, one individual had recurrent cutaneous abscesses, suggesting a possible link to sustained colonization.

## Introduction

The transmission of pathogenic agents among hospitalized patients poses a significant challenge for infection control programs. Colonized individuals face a heightened risk of developing infections, making it crucial to understand the dynamics of such transmissions. *Staphylococcus Aureus* (SA), a common human commensal bacterium, transiently colonizes the nasal cavities and skin of 30 %‒60 % of the population, with 20 % persistently colonized.^1^^,^^2^ Methicillin-Resistant *Staphylococcus Aureus* (MRSA) complicates this scenario by developing resistance to beta-lactam antibiotics through the synthesis of an additional Penicillin-Binding Protein (PBP2a), encoded by the *mec*A gene^3^ MRSA is notorious for causing outbreaks in hospitals and has become a significant concern in community settings as well.

Over the past few decades, community-acquired MRSA (CA-MRSA) has emerged, characterized by the production of Panton-Valentine Leukocidin (PVL) and the presence of *SCCmec* types IV and V. Transmission of MRSA primarily occurs through direct contact or contaminated objects^4^. The bacteria can survive on surfaces such as gloves, cotton towels, and medical equipment for extended periods,^5^ exacerbating the challenge of controlling its spread. In hospital environments, stringent hygiene practices and isolation protocols are essential to prevent outbreaks, but the dynamics of MRSA transmission within households remain less clear.

Preventing interpersonal transmission in community settings requires avoiding direct contact and the exchange of objects, which is challenging due to the shared nature of personal items and communal areas.^6^^,^^7^ The literature on household MRSA transmission and colonization patterns shows a lack of consensus, complicating prevention efforts^8^, ^9^. This study aims to evaluate the persistence of MRSA colonization in asymptomatic carriers identified in a previous study^10^ and to determine whether other household members are colonized, providing further insights into the intra-household transmission dynamics of MRSA.

## Methods

We evaluated household colonization in seven families that had a member previously identified as MRSA-colonized from a community study^10^. All household members of the MRSA-colonized patient were included in the study to provide a comprehensive assessment of intra-household transmission. To gather relevant personal data, previous medical history, and antibiotic use over the past year, a detailed questionnaire focusing on recognized risk factors for MRSA transmission was administered to each participant.

To collect nasal samples from each subject, we utilized COPAN Venturi Transystem® swabs (COPAN Diagnostics Inc., Murrieta, California, USA). These swabs were inoculated in Brain Heart Infusion medium (BHI) with 2.5 % NaCl, incubated for 24 h, and then seeded on regular mannitol agar. Samples that converted mannitol agar to yellow were identified using MALDI-TOF MS Microflex™ (Bruker Daltonics, Billerica, Massachusetts, USA). *Staphylococcus Aureus* (SA) isolates underwent antimicrobial susceptibility testing by microdilution for oxacillin^11^, with resistant isolates confirmed by PCR for the presence of *mec*A and *co*A genes.

For the MRSA isolates from the previous study,^10^ we conducted Whole-Genome Sequencing (WGS) using Illumina technology to characterize resistance and virulence profiles. Genome annotation was performed with Prokka v.1:11, and Sequence Typing (ST) was confirmed using the MLSTfinder tool and the PubMLST database. Additionally, PCR multiplex for *SCCmec* type was conducted using specific primers and conditions to further classify the MRSA strains based on their genetic elements.

Data analysis involved comparing the new samples with those from the previous study to evaluate spontaneous clearance and intra-household transmission of MRSA. The study was approved by the Research Ethics Committees (number), ensuring adherence to ethical standards and maintaining data confidentiality throughout the research process. This comprehensive approach allowed us to investigate the persistence and transmission dynamics of MRSA within households, providing valuable insights into its epidemiology.

## Results

We collected 25 samples from seven families 17 to 20 months after the initial sample collection. Of these samples, 56 % (14) were identified as *S. aureus* and 44 % (11) were non-*S. aureus*, confirmed by MALDI-TOF. Among the *S. aureus* isolates, four exhibited methicillin resistance with a Minimum Inhibitory Concentration (MIC) of ≥ 4 μg/mL and were PCR positive for both *co*A and *mec*A genes, as detailed in Table 1. Notably, three families displayed limited intra-household MRSA transmission, with only one family (Family 3) having all members colonized with MRSA. These members shared the same *SCCmec* type and resistance profile, indicating a common source of transmission within the household. Furthermore, six of the seven index cases had lost MRSA colonization by the time of the second sample collection.

**Table 1:** Summary of Methicillin-Resistant *Staphylococcus Aureus* (MRSA) colonization and follow-up results in seven families over 17‒20 months.

Family/Service	Index Case (MRSA)	Follow-Up	Household Members	Notes
**Family 1** Dermatology 2 people	Female, 66yo, WGS: ant(6)-Ia-like aph(3′)-III mecA mph(C) msr(A)-like norA-like SCCmec IVa ST-1176	17-months later, coag-negative	Husband: coag-negative	Husband hospitalized for urinary sepsis, multiple antibiotics uses (Amoxicillin, Ciprofloxacin, Ertapenem)
**Family 2** HU-USP 5 people	Male, 24yo, WGS: blaZ mecA norA-like SCCmec V ST-05	17-months later, *S. aureus* (MSSA)	Father: coag-neg Mother: SA Brother: SA Girlfriend: SA, (MRSA positive URC)	Family uses antiseptic soaps and mouthwashes. Various antibiotic uses including Amoxicillin, Ciprofloxacin, Cephalexin
**Family 3** Dermatology 2 people	Male, 34yo, WGS: ant(6)-Ia-like aph(3′)-III blaZ-like mecA mph(C) msr(A)-like norA-like SCCmec IVa ST-08 PVL+	17-months later, MRSA (coA/mecA) SCCmec IVa	Wife: MRSA (coA/mecA) SCCmec IVa	Wife has recurrent furunculosis and anemia, used antibiotics (Amoxicillin, Ciprofloxacin), underwent myomectomy
**Family 4** HC-FMUSP 5 people	Female, 47yo, WGS: ant(6)-Ia-like aph(3′)-III blaZ-like mecA mph(C) msr(A)-like norA-like SCCmec II/V ST-08 PVL+	19-months later, *S. aureus* (MSSA)	Husband: MRSA mic:4; Daughter: S. haemolyticus; Daughter: SA Daughter: coag-neg	All members have skin diseases. Various antibiotic uses and medical conditions
**Family 5** Dermatology 4 people	Female, 51yo, WGS: ant(6)-Ia-like aph(3′)-III mecA mph(C) msr(A)-like norA-like SCCmec IVa ST-1176	17-months later, coag-negative	Father: coag-neg Husband: MRSA Brother: SA	No additional specific family details provided
**Family 6** Dermatology 2 people	Male, 21yo, WGS: blaZ mecA norA-like ST-06	18 months later, S. aureus (MSSA)	Mother: coag-neg	No particularities noted
**Family 7** Dermatology 5 people	Female, 42yo, WGS: aadD-like mecA mph(C) msr(A)-like tet(K) SCCmec: IVa	20 months later, coag-negative	Son: SA; Daughter-in-law: coag-neg; Grandson: S. haemolyticus; Son: SA	Multiple family members underwent surgeries, grandson used Amoxicillin for tonsillitis, no skin diseases reported

MRSA, Methicillin-Resistant *Staphylococcus Aureus*; coA, Coagulase gene; mecA, Methicillin resistance gene; WGS, Whole Genome Sequencing; SCCmec, Staphylococcal Cassette Chromosome mec; ST, Sequence Typing; PVL, Panton-Valentine Leukocidin; MSSA, Methicillin-Sensitive *Staphylococcus Aureus*; ant(6)-Ia, Aminoglycoside Nucleotidyltransferase gene variant; aph(3′)-III, Aminoglycoside Phosphotransferase gene; mph(C), Macrolide Phosphotransferase gene; msr(A), Macrolide Efflux Protein gene; norA, Norfloxacin resistance protein gene; blaZ, Beta-lactamase gene; aadD, Aminoglycoside Adenylyltransferase gene; tet(K), Tetracycline resistance protein gene; SA, *Staphylococcus Aureus*; URC, Urine Routine Culture; ATB, Antibiotics; HAS, Hypertension (Hypertensão Arterial Sistêmica); DM2, Diabetes Mellitus Type 2.

In the three new MRSA cases (Families 3, 4, and 5), all individuals reported antibiotic use over the past year, particularly beta-lactams. This suggests a possible link between antibiotic use and the emergence of new MRSA colonization. Additionally, three individuals had undergone acupuncture, which may have contributed to their MRSA colonization. A history of skin disease was noted in all families with MRSA cases, with at least one member in each family having experienced a skin condition. This commonality highlights skin disease as a potential risk factor for MRSA colonization and transmission within households.

These findings underscore the complexity of MRSA transmission dynamics within households. The loss of MRSA colonization in some individuals over time suggests that MRSA colonization can be transient. However, the identification of new MRSA cases and the association with antibiotic use and skin disease indicate ongoing risks and the need for targeted infection control measures. The study's results highlight the importance of continuous monitoring and tailored interventions to prevent MRSA spread in community settings.

## Discussion

This study demonstrates a high rate of spontaneous MRSA colonization clearance, with six of the seven original cases no longer colonized after 17‒20-months. This finding aligns with previous studies, which have reported spontaneous clearance periods ranging from 4 to 36-weeks^5^. The clearance of MRSA colonization is influenced by various host factors, such as the individual's immune status and the virulence of the MRSA strain. The observed spontaneous clearance rate underscores the dynamic nature of MRSA colonization, where the body's natural defenses can eventually overcome the bacterial presence.

Our findings indicate limited intra-household MRSA transmission, as evidenced by the low number of colonized individuals per household. Notably, all families with MRSA cases had at least one member with a history of skin disease. The persistence of MRSA in one family may be linked to recurrent cutaneous abscesses in one individual, suggesting that disruptions in the normal skin microbiota can predispose individuals to MRSA colonization and infection^12^. This highlights the importance of skin health in preventing MRSA colonization and underscores the need for targeted interventions in individuals with chronic skin conditions.

Despite the small number of households studied and the non-standardized follow-up period, our observations provide valuable insights into MRSA clearance and its association with skin diseases. The heterogeneous nature of the sample population suggests that these findings may be generalizable to a broader community setting. Given the established link between chronic skin diseases and MRSA persistence, we recommend considering MRSA eradication therapy for patients with chronic skin conditions to reduce the risk of ongoing colonization and potential transmission.

In conclusion, this study highlights the dynamic nature of MRSA colonization and the significant role of skin health in influencing MRSA persistence and transmission. The high rate of spontaneous MRSA clearance observed reinforces the importance of monitoring and supporting immune health in colonized individuals. Targeted interventions, particularly for those with chronic skin conditions, could play a crucial role in reducing MRSA colonization and preventing intra-household transmission. Further research with larger sample sizes and standardized follow-up periods is necessary to validate these findings and develop comprehensive MRSA management strategies.

## Conflicts of interest

The authors declare no conflicts of interest.
